# Fluorescence imaging of ATP in neutrophils from patients with sepsis using organelle-localizable fluorescent chemosensors

**DOI:** 10.1186/s13613-016-0175-z

**Published:** 2016-07-15

**Authors:** Koichiro Sueyoshi, Yuka Sumi, Yoshiaki Inoue, Yoko Kuroda, Kumiko Ishii, Hitoshi Nakayama, Kazuhisa Iwabuchi, Yasutaka Kurishita, Hajime Shigemitsu, Itaru Hamachi, Hiroshi Tanaka

**Affiliations:** Department of Emergency and Critical Care Medicine, Juntendo University, Urayasu Hospital, 2-1-1 Tomioka, Urayasu, Chiba 279-0021 Japan; Lipid Biology Laboratory, Riken Advanced Science Institute, Wako, Saitama Japan; Institute for Environmental and Gender Specific Medicine, Juntendo University, Urayasu, Chiba Japan; Department of Synthetic Chemistry and Biological Chemistry, Graduate School of Engineering, Kyoto University, Kyoto, Japan

**Keywords:** Polymorphonuclear neutrophils (PMNs), PMAP-1, MitoAP-1, Plasma membrane, Mitochondria, Flow cytometry

## Abstract

**Background:**

The activation of polymorphonuclear neutrophils (PMNs) plays an important role in sepsis. Previously, we showed that ATP release and feedback via ATP receptors are essential for PMN activation; however, the dynamics remain poorly understood. Two new fluorescent chemosensors, PMAP-1 and MitoAP-1, were developed to detect ATP in the plasma membrane and mitochondria of living cells, respectively. In this study, we aimed to evaluate ATP localization using these chemosensors in PMNs of sepsis patients.

**Methods:**

Live PMNs isolated from 16 sepsis patients and healthy controls (HCs) were stained with these chemosensors and observed by confocal microscopy, and their mean fluorescence intensities (MFIs) were evaluated using flow cytometry. CD11b expression in PMNs was also evaluated.

**Results:**

The MFIs of PMAP-1 and MitoAP-1 and CD11b expression in PMNs from sepsis patients on days 0–1 were significantly higher than those of HCs. The MFI of PMAP-1 and CD11b expression on days 3–4 decreased significantly compared to those observed at days 0–1, whereas MitoAP-1 MFI was maintained at a high level. The PMAP-1 MFI was significantly positively correlated with CD11b expression, white blood cell counts, neutrophil counts, and C-reactive protein levels in patients.

**Conclusions:**

The higher MFIs of PMAP-1 and MitoAP-1 in sepsis patients suggest a pivotal role of ATP for PMN activation. The temporal difference in ATP levels suggests that ATP plays different roles in the mitochondria and on the cell surface. These data should contribute to the understanding of the dynamics of ATP in PMNs and help to develop a novel therapy for sepsis.

## Background

ATP is well known as the energy currency of cells and is involved in both intracellular signaling and extracellular signaling. Although polymorphonuclear neutrophils (PMNs) are one of the most important phagocytic cell types in defending against invading pathogens and play a pivotal role in inflammation, their activation can be a double-edged sword. In particular, their pathophysiological processes can damage the host tissue when activated during sepsis, trauma, ischemia–reperfusion injury, and inflammatory diseases [1]. PMNs can migrate to compromised tissues in response to chemotactic gradients. The activation of PMNs is triggered by the recognition of bacterial peptides and innate inflammatory mediators. Although a signal amplification system is crucial for the activation of PMNs, the underlying mechanisms remain obscure [2–4]. We previously found that ATP was released extracellularly by PMNs through pannexin-1 (PANX1) hemichannels and that autocrine feedback through the P2Y2 receptor, one of eight P2Y G protein-coupled receptors, is an essential mechanism for activating PMNs [5, 6]. We also reported that plasma ATP levels were correlated with PMN activation and were significantly higher in mouse models of cecal ligation and puncture (CLP) [7].

In recent decades, much effort has been dedicated to developing fluorescent chemosensors for ATP to study its diverse functions [8, 9]. Consequently, two new fluorescent chemosensors were developed (PMAP-1 and MitoAP-1), which can detect ATP and its derivatives in specific regions of living cells [10]. PMAP-1 and MitoAP-1 are also referred to as 2-2Zn(ΙΙ) and 3-2Zn(ΙΙ), respectively [10]. PMAP-1 can localize to the plasma membrane and detect the extracellular release of ATP. In contrast, MitoAP-1 can spontaneously localize in the mitochondria and detect local ATP.

Sepsis is the major cause of death in critically ill patients and is the predominant cause of multiple organ dysfunction syndrome [11, 12]. In spite of progress in intensive care medicine and strict adherence to established treatment protocols such as the “Surviving Sepsis Campaign” guidelines [13], mortality in sepsis patients remains high. Millions of patients worldwide are afflicted each year, resulting in the death of one in three patients [11]. Multiple organ failure in sepsis is the prevailing cause of death, and mitochondrial dysfunction has been suggested to contribute to the development of organ dysfunction and failure in sepsis [14]. However, the associated pathogenic mechanisms remain unclear.

In this study, we evaluated a new approach to quantify ATP release on the cell surface and ATP production in the mitochondria of PMNs by flow cytometry. As described above, ATP is known to play a key role in the activation of PMNs; thus, we hypothesized that ATP levels on the plasma membrane and in the mitochondria of PMNs could serve as an informative biomarker for sepsis. Here, we report the correlation between ATP levels and the clinical conditions of sepsis patients.

## Methods

### Patients

After obtaining approval from the ethics committee of Juntendo University, Urayasu Hospital (25–47), 16 sepsis patients admitted to our critical care center between October 2013 and April 2015 were enrolled in the study. All patients met the diagnostic criteria for sepsis determined by the American College of Chest Physicians and Society of Critical Care Medicine [15], and all patients (or their legal representatives) provided informed consent for participation in the research. Patients with malignant disease or chronic administration of steroids were excluded. The patients comprised nine males and seven females, with a mean ± SD age of 67 ± 9 years, a serum C-reactive protein (CRP) level of 27.0 ± 12.1 mg/dL, a white blood cell (WBC) count of 13.3 ± 9.1 × 10^3^/μL, an Acute Physiology and Chronic Health Evaluation (APACHE) II score [16] of 22.1 ± 7.8, a Sequential Organ Failure Assessment (SOFA) score [17] of 7.9 ± 4.6, and a Japanese Association for Acute Medicine (JAAM) disseminated intravascular coagulation (DIC) score [18] of 3.8 ± 1.7. The cause of sepsis was urinary tract infection in five patients; burn in three patients; pneumonia in three patients; and necrotic cholecystitis, cellulitis, gas gangrene, cervical abscess, and peritonitis in one patient each. Two patients died during hospitalization (Table 1). The healthy control (HC) group consisted of six males and three females, with a mean ± SD age of 37 ± 5 years.

**Table 1:** Characteristics of sepsis patients

Patients	Cause of sepsis	APACHE ΙΙ score	SOFA score	JAAM DIC score	WBC (/μL)	CRP (mg/dL)	Mortality
1.	Necrotic cholecystitis	31	15	7	4600	27.9	Alive
2.	Pneumonia	18	3	4	18,600	48.8	Alive
3.	Burn	12	2	2	10,600	23.9	Alive
4.	Pneumonia	24	7	3	17,900	35.8	Dead
5.	Cellulitis	11	3	3	19,300	54.0	Alive
6.	Burn	29	12	4	4100	14.2	Dead
7.	UTI	36	14	8	13,900	17.9	Alive
8.	UTI	26	11	3	16,400	15.5	Alive
9.	UTI	14	6	3	20,700	10.4	Alive
10.	Burn	29	13	4	4600	25.5	Alive
11.	UTI	20	8	6	31,900	25.8	Alive
12.	Gas gangrene	17	2	2	17,100	15.6	Alive
13.	Cervical abscess	20	5	3	5000	29.4	Alive
14.	UTI	11	4	3	800	29.7	Alive
15.	Peritonitis	29	9	3	23,700	21.8	Alive
16.	Pneumonia	27	13	3	900	36.0	Alive

*APACHE* Acute Physiology and Chronic Health Evaluation, *SOFA* Sequential Organ Failure Assessment, *JAAM* Japanese Association for Acute Medicine, *DIC* disseminated intravascular coagulation, *WBC* white blood cell counts, *CRP* C-reactive protein, *UTI* urinary tract infection

### Blood samples

A 10-mL heparinized blood sample was collected via the peripheral vein of each healthy volunteer and via arterial lines in sepsis patients. Blood collection for all sepsis patients was performed within 24 h after the patients were diagnosed with sepsis (days 0–1) and at days 3–4 of sepsis.

### Isolation of PMNs

Human PMNs were isolated from the heparinized whole blood of healthy volunteers and sepsis patients using Polymorphprep™ centrifugation (Alere Technologies AS, Oslo, Norway). The neutrophil population was >95 % pure, as determined by Wright–Giemsa staining.

### PMAP-1 and MitoAP-1

Both PMAP-1 and MitoAP-1 were designed on the basis of a fluorescent ATP probe containing a xanthene fluorophore [10]. PMAP-1 selectively localizes on the plasma membrane via a biocompatible anchor for the membrane, which possesses a hydrophobic oleyl moiety at the end of a long ethylene glycol linker [10]. MitoAP-1 selectively localizes to the mitochondria via a positively charged pyronin ring [10].

### Imaging of PMNs with confocal microscopy

Isolated human PMNs (2.0 × 10^6^ cells/mL) were plated on a 35-mm glass-bottomed dish (IWAKI, Tokyo, Japan), coated with 100 μL of poly-l-lysine solution (Sigma-Aldrich, St. Louis, MO, USA), and incubated for 10 min. All incubations were performed under a humidified atmosphere of 5 % CO_2_ at 37 °C in the dark. Cells were treated with 400 nM (final concentration) of MitoTracker^®^ Green FM (Invitrogen, Carlsbad, CA, USA), followed by incubation with 1 μM MitoAP-1 (final concentration) for 20 min. Cells were incubated with 5 μM PMAP-1 (final concentration) for 10 min. After washing twice with 37 °C Hanks’ balanced salt solution (HBSS, Sigma-Aldrich), stained cells were examined with a TCS SP5 Leica confocal laser-scanning microscope equipped with a Plan-Apochromat 100× oil-immersion objective lens at 37 °C, under a 5 % CO_2_ humidified atmosphere.

### Fluorescence quantification by flow cytometry

Isolated PMNs were washed with HBSS and incubated at 37 °C for 10 min with or without *N*-formyl-met-leu-phe (fMLP, ScyTek Laboratories, Logan, UT, USA) stimulation before staining. Cells were incubated with 1 μM (final concentration) of MitoAP-1 and allophycocyanin-conjugated anti-CD11b antibodies (BD Biosciences, San Jose, CA, USA) at 37 °C for 20 min, followed by incubation with 5 μM PMAP-1 (final concentration) at 37 °C for 10 min. The cells were placed on ice for 10 min and centrifuged at 1500 rpm for 5 min at 4 °C. After washing twice with HBSS, cells were fixed with fluorescence-activated cell sorting (FACS) sheath fluid (BD Biosciences) and then analyzed by flow cytometry (FACSCalibur, BD Bioscience).

### Statistical analysis

The data are expressed as the mean ± SEM unless stated otherwise. Statistical analyses were performed using GraphPad Prism version 6.0 (GraphPad, San Diego, CA, USA). The data were analyzed for significant differences by a paired *t* test or Wilcoxon signed-rank test for comparisons of paired groups. One-way analysis of variance (ANOVA) and the Kruskal–Wallis test were used for comparisons of three groups. Spearman’s rank correlation was performed as indicated. Differences were considered statistically significant at *p* < 0.05.

## Results

### Successful establishment of a staining protocol for human PMNs with MitoAP-1

**Fig. 1:**
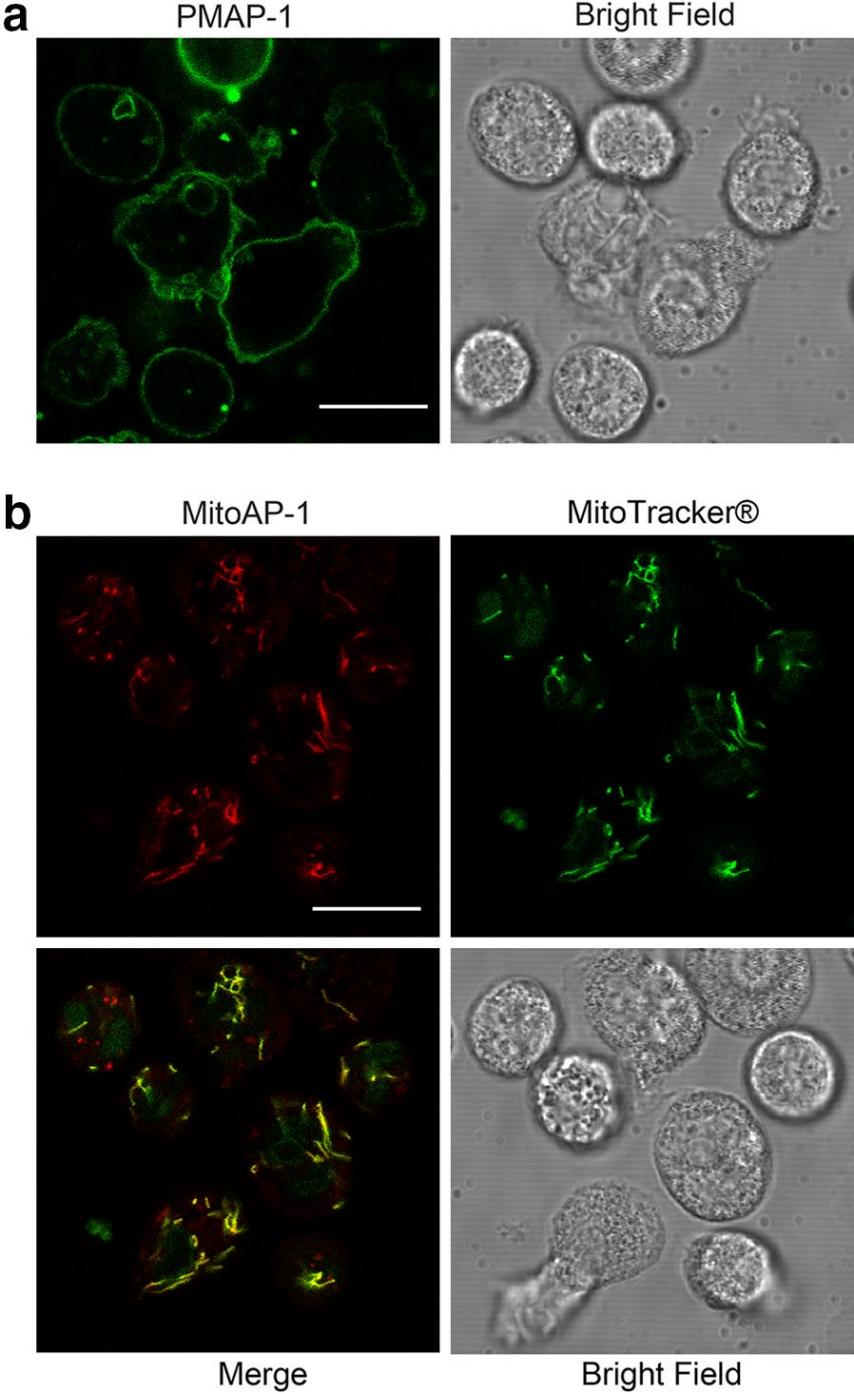
Confocal micrographs of human polymorphonuclear neutrophils (PMNs) stained with PMAP-1 and MitoAP-1. **a** ATP on the plasma membrane was stained with PMAP-1. The *bright green* fluorescence observed on the plasma membrane of PMNs resulted from PMAP-1 conjugation to ATP (×100 oil objective, NA 1.4). *Scale bar* 10 μm. **b** ATP in the mitochondria was stained with MitoAP-1 (*red*) and MitoTracker^®^ Green FM (*green*), and they colocalized well (×100 oil objective, NA 1.4). *Scale bar* 10 μm

Imaging studies using PMAP-1 and MitoAP-1 were limited to cultured cells [10] and T cells [19] until Bao et al. [20] first reported imaging studies of human PMNs using PMAP-1. As in their study, the bright green fluorescence emitted by PMAP-1 was predominantly observed on the plasma membrane of PMNs (Fig. 1a). However, there has been no previous report of the successful staining of human PMNs with MitoAP-1. We observed a punctate pattern of bright red fluorescence emitted by MitoAP-1 within the cells. MitoAP-1 fluorescence overlapped well with that of MitoTracker^®^ Green FM, a typical dye used to stain the mitochondria, indicating that MitoAP-1 selectively and spontaneously localized to the mitochondria (Fig. 1b). Thus, we successfully established a method for staining human PMNs with MitoAP-1.

### ATP release on the plasma membrane of HC PMNs increased with fMLP stimulation

**Fig. 2:**
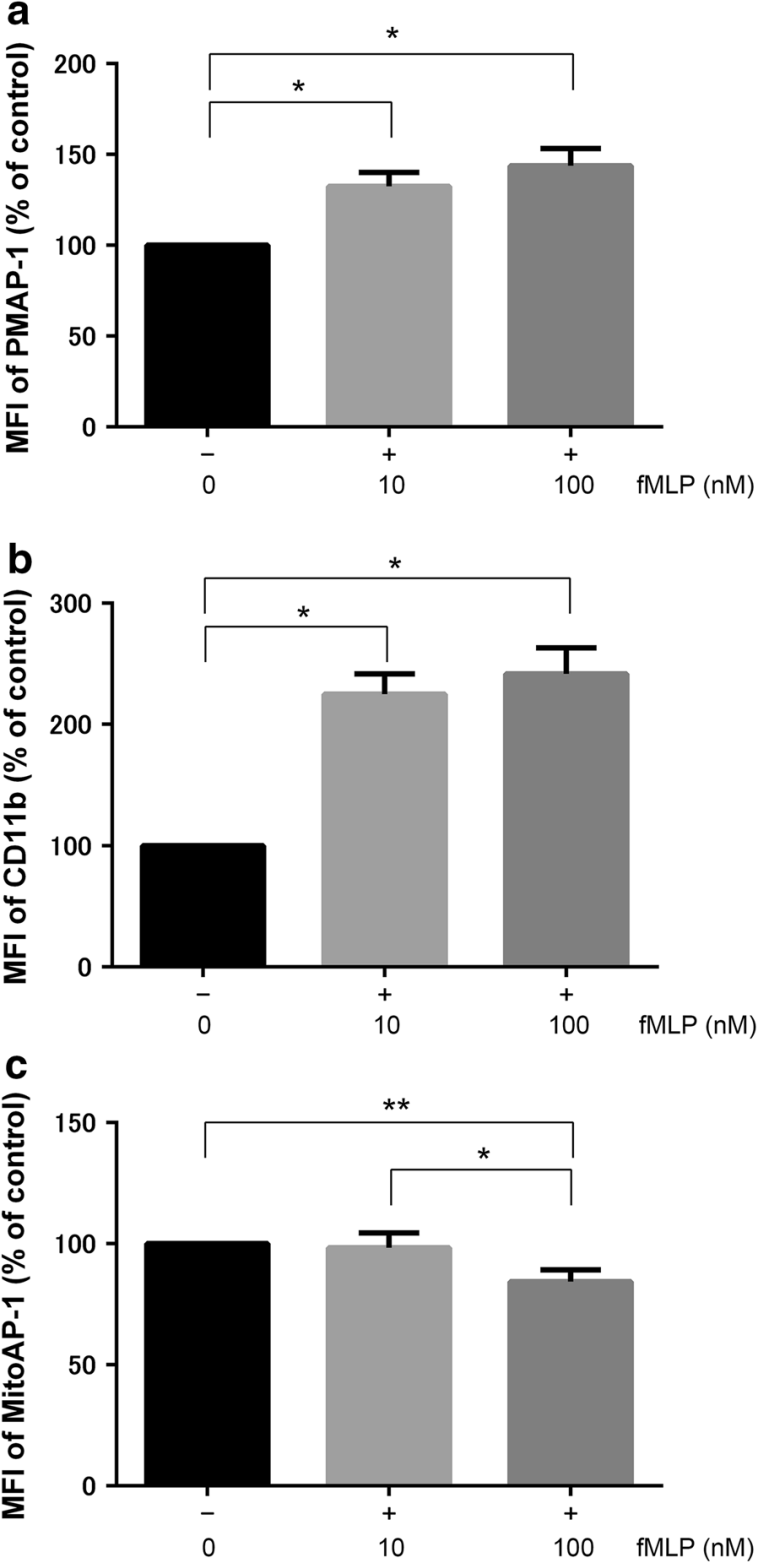
ATP level and CD11b expression after fMLP stimulation in healthy control subjects. **a** Changes in the mean fluorescence intensity (MFI) of PMAP-1 after stimulation with the indicated fMLP concentrations; MFI values were normalized to those of controls (no fMLP) (*n* = 8 per group). The data shown are the mean ± SEM, and groups were compared with one-way ANOVA and Tukey’s post hoc test (*p < 0.01). **b** Changes in CD11b expression of polymorphonuclear neutrophils after fMLP stimulation; expression levels were normalized to those of controls (no fMLP) (*n* = 8 per group). The data shown are the mean ± SEM, and groups were compared with one-way ANOVA with Tukey’s post hoc test (*p < 0.01). **c** Changes in MFI of MitoAP-1 after stimulation with the indicated fMLP concentrations; MFI values were normalized to those of controls (no fMLP) (*n* = 8 per group). The data shown are the mean ± SEM, and groups were compared with one-way ANOVA with Tukey’s post hoc test (*p < 0.01, **p < 0.05)

Stimulation of the formyl peptide receptor was previously found to cause ATP release from PMNs through maxi-anion channels and PANX1 hemichannels [6]. To confirm this finding, the MFI of PMAP-1 on the plasma membrane of HC PMNs was quantified by flow cytometry after stimulation with the indicated concentrations of fMLP (Fig. 2a). The MFI of PMAP-1 following fMLP stimulation increased in a dose-dependent manner. CD11b expression on the plasma membrane of PMNs was also measured as a marker of PMN activation. Cell surface CD11b expression in PMNs stimulated with fMLP also increased in a dose-dependent manner (Fig. 2b).

### Mitochondrial ATP levels in the HC group decreased with fMLP stimulation

Mitochondria are often referred to as the powerhouse of the cells, as they generate ATP by oxidative phosphorylation. To our knowledge, changes in mitochondrial ATP levels in PMNs have not been reported. The MFI of MitoAP-1 following fMLP stimulation decreased significantly in a dose-dependent manner (Fig. 2c).

### ATP release on the plasma membrane and mitochondrial ATP levels in PMNs were higher in sepsis patients than in HC subjects

**Fig. 3:**
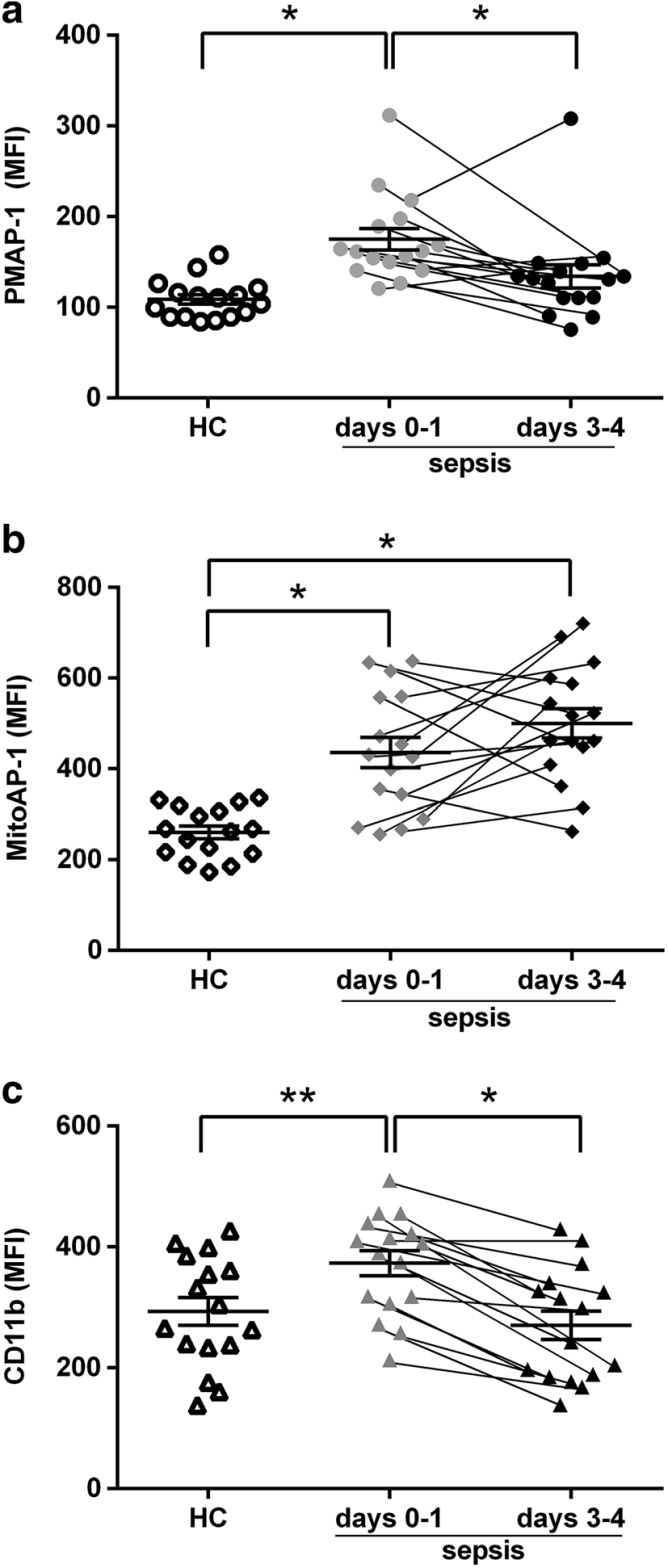
Mean fluorescent intensity (MFI) of PMAP-1, MitoAP-1, and CD11b expression in PMNs in sepsis patients and healthy control (HC) subjects. **a** MFI of PMAP-1 in sepsis patients (days 0–1 postdiagnosis) was significantly higher than that of HCs and decreased significantly on days 3–4 (mean ± SEM, Kruskal–Wallis test with Dunn’s post hoc test, *p < 0.01). **b** The MFI of MitoAP-1 in sepsis patients (days 0–1) was significantly higher than that of HCs and did not decrease significantly on days 3–4 (mean ± SEM, Kruskal–Wallis test with Dunn’s post hoc test, *p < 0.01). **c** CD11b expression in sepsis patients (days 0–1) was significantly higher than that of HCs and decreased significantly on days 3–4 (mean ± SEM, one-way ANOVA with Tukey’s post hoc test, *p < 0.01, **p < 0.05)

The MFIs of PMAP-1 and MitoAP-1 in sepsis patients were evaluated by flow cytometry within 24 h after diagnosis of sepsis (days 0–1). Both values were significantly higher than those of the HC group (Fig. 3a, b). CD11b expression on the plasma membrane was also upregulated in sepsis patients (Fig. 3c). Activated PMNs in sepsis patients appeared to cause a burst of extracellular ATP release and to increase ATP synthesis by oxidative phosphorylation in the mitochondria. The same experiments were conducted at days 3–4 as the patients’ clinical conditions improved, which revealed that the MFIs of PMAP-1 and CD11b expression decreased significantly compared to those at days 0–1 (Fig. 3a, c), whereas a high MitoAP-1 MFI was maintained (Fig. 3b). Namely, the temporal changes of ATP release and CD11b expression on the plasma membrane were similar, but ATP production showed distinct behavior in the mitochondria.

### ATP release on the plasma membrane correlated with PMN activation

**Fig. 4:**
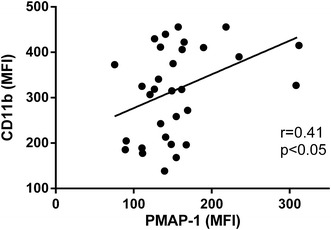
Correlation between the mean fluorescent intensity (MFI) of PMAP-1 and polymorphonuclear neutrophil (PMN) activation (CD11b expression). Comparison of PMAP-1 and CD11b expression on the plasma membrane showed a significant positive correlation in sepsis patients (*n* = 16, Spearman’s rank correlation coefficient, *p* < 0.05). The 32 data points shown consist of samples from both days 0–1 and days 3–4 following sepsis diagnosis

As described above, ATP release and CD11b expression decreased as the clinical conditions of the patients improved. Furthermore, the MFIs of PMAP-1 and CD11b expression showed a significant positive correlation in the sepsis patients (Fig. 4).

### ATP release on the plasma membrane correlated with WBC counts, neutrophil counts, and CRP levels

**Fig. 5:**
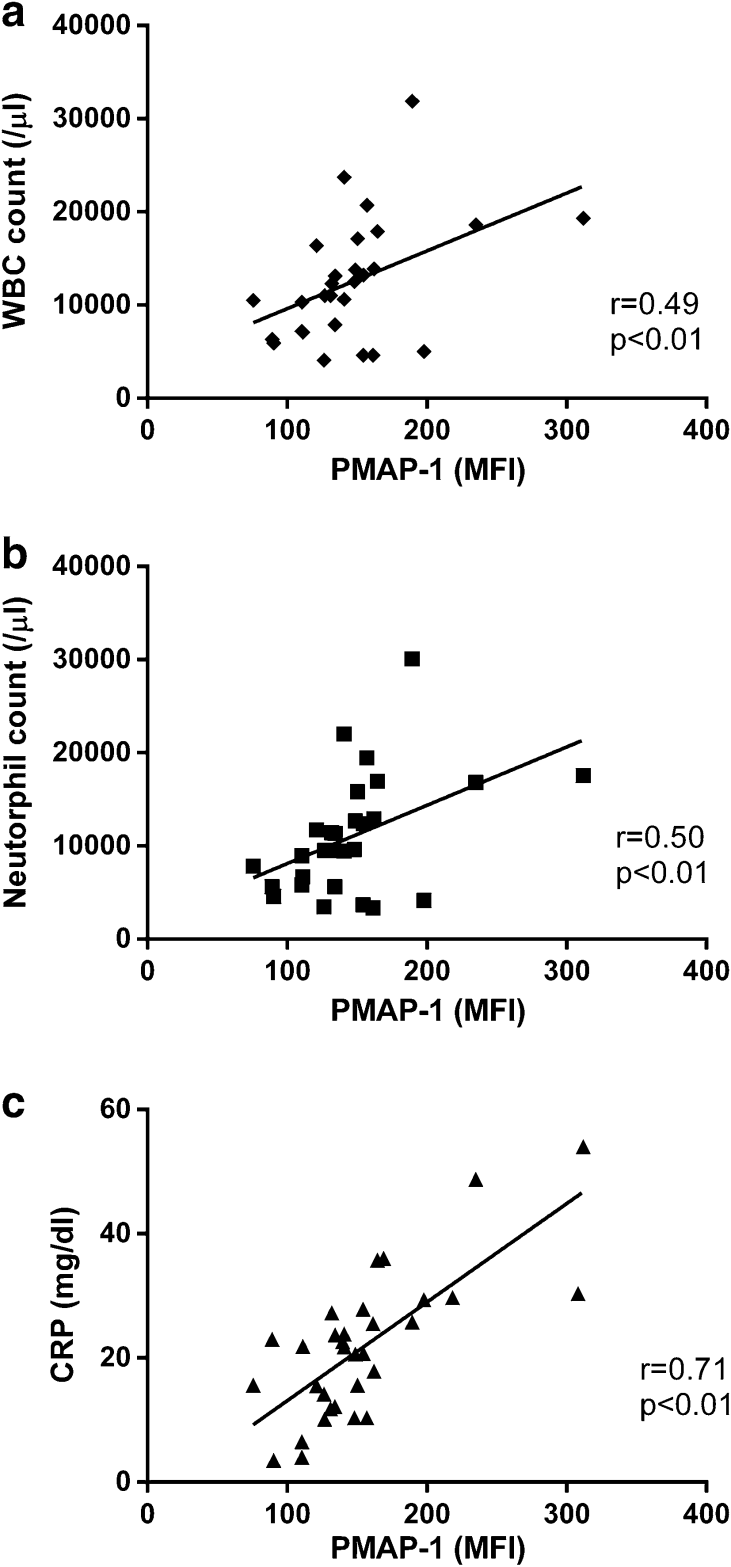
Correlation of PMAP-1 MFI with white blood cell (WBC) counts, neutrophil counts, and C-reactive protein (CRP) levels in sepsis patients. **a**, **b** Spearman’s rank correlation coefficient, *p* < 0.01 (*n* = 14). Two patients with leukopenia were excluded. The 28 data points shown consist of samples from both days 0–1 and days 3–4 following sepsis diagnosis. **c** Spearman’s rank correlation coefficient, *p* < 0.01 (*n* = 16). The 32 data points shown consist of samples from both days 0–1 and days 3–4 of sepsis

To determine relationships between ATP release on the plasma membrane or ATP production in the mitochondria and clinical conditions (WBC counts, neutrophil counts, CRP levels, SOFA scores, APACHE II scores, and JAAM DIC scores), their correlation coefficients were investigated. The MFI of PMAP-1 was significantly positively correlated with WBC counts, neutrophil counts, and CRP levels (Fig. 5), but not with SOFA scores, APACHE II scores, and JAAM DIC scores. No correlation was found between the MFI of MitoAP-1 of PMNs and any clinical variable.

## Discussion

To our knowledge, this is the first report of the visualization of mitochondrial ATP in the PMNs of sepsis patients with fluorescent chemosensors. PMNs are the major leukocytes recruited to inflamed sites in response to infection. Although PMNs play a central role in protecting the host from infection, their pathophysiological processes can also cause tissue damage in the host [1, 21]. To develop therapies that can reduce PMN-induced tissue damage, it is crucial to elucidate the mechanisms underlying the activation of PMNs.

ATP, which is well known as the “molecular unit of currency” of intracellular energy transfer, is released extracellularly by physiological stimulation and acts as a signaling molecule. Burnstock et al. [22] first reported that healthy neurons can release cellular ATP, which then serves as an intercellular messenger. Recently, neurons and many other types of cells have been shown to release ATP under physiological stimulation, including PMNs [23–33]. Previously, we showed that ATP release and purinergic signaling are essential for PMN activation. Extracellular ATP is released by PANX1 hemichannels and controls the chemotaxis of PMNs through P2Y2 receptors [5, 6]. The autocrine feedback through P2Y2 receptors is essential for PMN activation [6].

Substantial effort has been dedicated to developing fluorescent chemosensors for ATP to elucidate its diverse physiological functions [8–10, 34, 35]. To date, a few imaging studies using genetically encoded fluorescence ATP probes have been reported for this purpose [34, 35]. These probes are posttranslationally conjugated to a protein anchored to a certain organelle through genetically encoded localization signals. Although successful, these techniques are not convenient, because they require cumbersome and time-consuming processes for protein expression and maturation. Therefore, Kurishita et al. [10] developed two new types of fluorescent chemosensors, PMAP-1 and MitoAP-1, which can spontaneously and selectively localize in a specific cellular region [10]. For example, PMAP-1 localizes to the plasma membrane and can be used to detect the extracellular release of ATP, whereas MitoAP-1 localizes to the mitochondria and senses mitochondrial ATP. Thus, these fluorescent chemosensors are highly suited for investigating the dynamics of ATP in biological events occurring in target cellular regions [10].

The finding that cell surface ATP release and CD11b expression in the HC group increased dose-dependently following fMLP stimulation is consistent with our previous finding that ATP is released by fMLP stimulation via PANX1 hemichannels [6]. In sepsis patients, extracellular ATP release in PMNs was elevated on days 0–1, corroborating our previous work in mouse sepsis models, wherein plasma ATP levels were elevated and contributed to PMN activation [7]. Collectively, these findings indicate that PMNs may be a main source of elevated circulating ATP levels. Furthermore, ATP release on the plasma membrane was correlated with CD11b expression, WBC counts, neutrophil counts, and CRP levels in sepsis patients; thus, ATP release on the plasma membrane may reflect PMN activation and could potentially serve as an activation marker for sepsis. Given that excessive activation of PMNs can damage host organs, inhibition of purinergic signaling could serve as a potential therapeutic strategy for treating sepsis. Previously, we reported that the P2 receptor antagonist suramin reduced PMN activation in a mouse CLP model [7]. Thus, suramin may potentially be used as an effective therapeutic strategy for sepsis patients. In contrast to the complications mediated by an overactive neutrophil compartment, severe systemic inflammation is a risk factor for the development of immune suppression. The circulating neutrophil pool displays a paradoxical phenotype, exhibiting characteristics of both activated and suppressed functionality. Therefore, further investigations are required to resolve this paradox of the role of PMNs in sepsis [36].

Mitochondria play an essential role in the activation of PMNs by producing ATP, which triggers the purinergic signaling processes [20]. During this process, mitochondria generate ATP and release ATP extracellularly following fMLP stimulation. However, in the HC group, the mitochondrial ATP levels in PMNs decreased with fMLP stimulation in a dose-dependent manner, demonstrating that the mitochondrial ATP was consumed in this process in healthy PMNs. Mitochondrial ATP levels in PMNs from sepsis patients were significantly higher than those of the HC group. Although further investigations are required to determine the definite cause for this difference, it is intriguing to speculate that pathways other than fMLP stimulation may be essential for ATP elevation in the mitochondria. Evaluation of mitochondrial function in various cells has been previously reported in the context of sepsis [37, 38]. For example, Adrie et al. [37] demonstrated reduced mitochondrial membrane potential in peripheral blood monocytes from patients with severe sepsis. On the other hand, Belikova et al. [38] showed peripheral blood mononuclear cells from patients with severe sepsis had a significantly higher baseline value of oxygen consumption compared with healthy volunteer cells. This report supports our study that mitochondrial ATP production in PMNs from sepsis patients was increased. Therefore, understanding the interaction among cells is essential to elucidate the mechanism of sepsis. Our method shows potential to clarify the mechanism of sepsis from the point of view of mitochondrial function.

Furthermore, although the PMAP-1 MFI on days 3–4 of sepsis decreased in parallel with CD11b downregulation, the MFI of MitoAP-1 did not change. These findings suggest that ATP serves different functions on the cell surface and in the mitochondria of PMNs in sepsis patients.

## Conclusions

In conclusion, we developed a novel approach for quantifying ATP release on the cell surface and mitochondrial ATP production in PMNs with two new types of fluorescent chemosensors, which was applied to the study of sepsis patients. ATP release on the plasma membrane of PMNs was demonstrated to be a potentially valuable biomarker for sepsis. Taken together with our previous studies, our current findings suggest that PMN responses can be regulated by therapeutic strategies that target the purinergic signaling mechanisms of PMNs. Additional studies are needed to determine whether and how these purinergic signaling mechanisms can be targeted in order to best benefit sepsis patients.

## Abbreviations

ANOVA: analysis of variance
APACHE: Acute Physiology and Chronic Health Evaluation
CLP: cecal ligation and puncture
CRP: C-reactive protein
DIC: disseminated intravascular coagulation
FACS: fluorescence-activated cell sorting
fMLP: *N*-formyl-met-leu-phe
HBSS: Hanks’ balanced salt solution
HC: healthy controls
JAAM: Japanese Association for Acute Medicine
MFI: mean fluorescence intensity
PMN: polymorphonuclear neutrophil
SOFA: Sequential Organ Failure Assessment
WBC: white blood cell
